# Pediatric laryngeal embryonal rhabdomyosarcoma^☆^

**DOI:** 10.1016/j.bjorl.2023.101291

**Published:** 2023-07-12

**Authors:** Bruna de Alencar Custodio Lupoli, Bárbara Paiva Mira, Carolina Sponchiado Miura, Elvis Terci Valera, Francesca Maia Faria, Gustavo Santos Boasquevisque, Fabiana Cardoso Pereira Valera

**Affiliations:** a Universidade de São Paulo, Hospital das Clínicas da Faculdade de Medicina de Ribeirão Preto, Departamento de Oftalmologia, Otorrinolaringologia e Cirurgia de Cabeça e Pescoço, Divisão de Otorrinolaringologia, Ribeirão Preto, SP, Brazil; b Universidade de São Paulo, Hospital das Clínicas da Faculdade de Medicina de Ribeirão Preto, Departamento de Pediatria, Divisão de Oncologia Pediátrica, Ribeirão Preto, SP, Brazil; c Universidade de São Paulo, Hospital das Clínicas da Faculdade de Medicina de Ribeirão Preto, Departamento de Patologia, Ribeirão Preto, SP, Brazil; d Universidade de São Paulo, Hospital das Clínicas da Faculdade de Medicina de Ribeirão Preto, Departamento de Imagens Médicas, Hematologia e Oncologia, Divisão de Radiologia, Ribeirão Preto, SP, Brazil

## Introduction

Rhabdomyosarcoma (RMS) is an aggressive malignant tumor, characterized by extensive local dissemination and by increased propensity to metastasis. It originates from embryonal mesenchymal cells that differentiate into skeletal muscle tissue. This is the most common sarcoma in childhood, representing more than 50% of the cases.^1^ Yet, the primary occurrence of this tumor in the larynx is quite unusual.

The symptoms and signs of RMS are variable, according to the region where the primary tumor originates, the age of the patient, and the presence or not of metastases. When the RMS is primarily located at the larynx, the most prominent symptoms are hoarseness, dysphagia, and dyspnea.

As therapy and prognosis depend on staging, clinical evaluation should address the extensiveness of the primary tumor and evaluate of the possible presence of metastases. In this regard, radiologic evaluation should include Computerized Tomography (CT) scans of the primary and adjacent tumor regions. Additional staging workup usually include chest CT scan, Magnetic Resonance Imaging (MRI) to address local dissemination and abdominal metastasis, and bone scintigraphy to evaluate bone metastasis. Bone marrow aspirate/biopsy are also used to evaluate bone marrow infiltration by the disease. A representative biopsy should be obtained to diagnose RMS, especially when primary radical surgeries may lead to morbidity or disfunctions. Definitive diagnosis occurs after histological analysis of the tumor, including immunohistochemistry panel.

Children with small, localized tumors of favorable histology and site, display very high cure rates. Yet, even with aggressive multimodal treatment, less than 20% of patients with metastatic disease are long-term survivals.^2^ Several prognostic factors for RMS have been reported. The main prognosticators accepted to date are primary tumor site and size, degree of resection, histological subtype, the presence of metastasis and lymph node involvement, fusions involving PAX3 and PAX7 genes and response to therapy.^3^

## Case report

A 5-year-old female patient was urgently referred for an otorhinolaryngological evaluation at our hospital due to progressive hoarseness and snoring for 8-months. Her respiratory pattern worsened a few days before admission, evolving to respiratory distress on mild exertion, dyspnea, dysphagia, and choking. She had undergone a laryngeal surgery previously at another hospital due to “laryngeal papillomatosis’’, but had early recurrence of symptoms.

At admission, she presented with hoarseness, dyspnoea, cyanosis in lips and malnutrition. No adenomegaly was found at the head and neck region.

**Figure 1:**
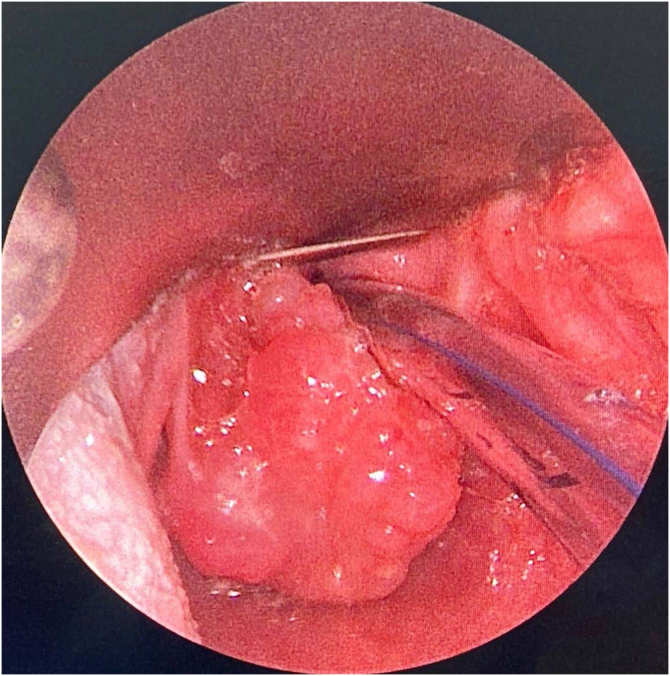
Solid tumor at the left supraglottic region at the laryngoscopy, when the patient was first admitted at the hospital.

Because of such extreme clinical condition, she was submitted to a laryngeal endoscopic surgery at once, as an emergency procedure. The intra-operative finding was the presence of a nodular lesion, involving the left poster-lateral area of supraglottic region; the consistency was solid and infiltrative (Fig. 1). After the lesion was partially removed, the patient remained without any respiratory discomfort.

**Figure 2:**
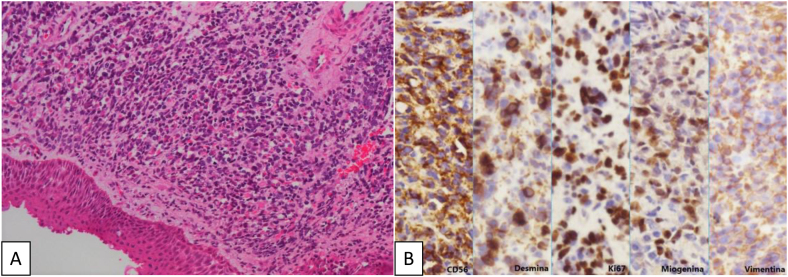
Histopathology (A) tumor with a diffuse pattern of infiltration (H&E, enhanced 200×); (B) immunohistochemistry panel: the tumor showed positive myogenin, desmin, vimentin and CD56, and high index of cell proliferation (KI67 + 80%) (Enhanced 400×).

Histopathologic exam showed a diffuse infiltrative neoplasia, projecting papilloma outlines covered by squamous epithelium, with low reactional changes, composed by rounded cells with scarce eosinophilic cytoplasm with an eccentric or fusiform aspect, hyperchromatic and pleomorphic nuclei, and some images of mitosis and myxedematous stroma. Immunohistochemistry staining depicted positivity for desmin, vimentin myogenin and CD56; KI67 labeling was high (80% of tumor cells) (Fig. 2). The final diagnosis was of an embryonal rhabdomyosarcoma of the larynx.

**Figure 3:**
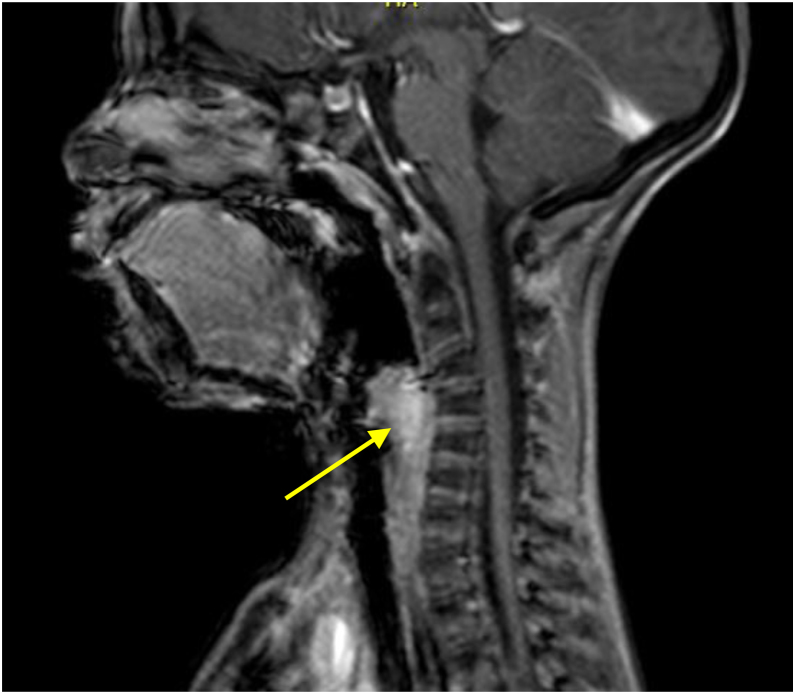
Cervical MRI, in sagittal plane. Arrow point to a well-defined nodular supraglottic expansive lesion, centered at the left aryepiglottic fold, presenting enhancement after contrast, without restriction to diffusion, measuring 2.5 × 1.8 × 2.4 cm.

**Figure 4:**
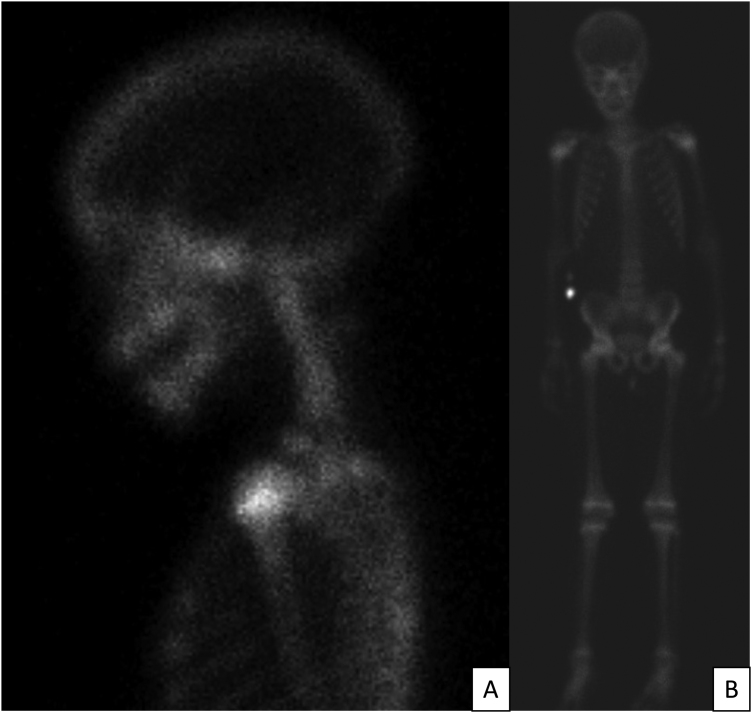
(A) detail in neck region; (B) Whole body bone scintigraphy showing normal distribution of the radiopharmaceutical, according to age and bone framework.

Contrast-enhanced CT scan and MRI showed an expansive and nodular lesion in supraglottis, well defined and centered at the left aryepiglottic fold. The lesion enhanced after contrast at CT, showed no restriction at diffusion at MRI, and measured 2.5 × 1.8 × 2.4 cm (Fig. 3). Full body scintigraphy with 99mTc-MDP showed no metastasis at the bones (Fig. 4).

The case was classified as Low Risk B according to European Pediatric Soft Tissue Sarcoma Study Group (EpSSG) Risk Classification, and chemotherapy was initiated — Brazilian Protocol (2011) (VAC/VCR — 45-week duration). Besides chemotherapy, the patient underwent local radiotherapy (IMRT — 28 fractions, with total dose of 50.4 Gy). Total resection of the lesion was scheduled to be performed after chemo and radiotherapy to avoid major functional complications and in quality of life.

**Figure 5:**
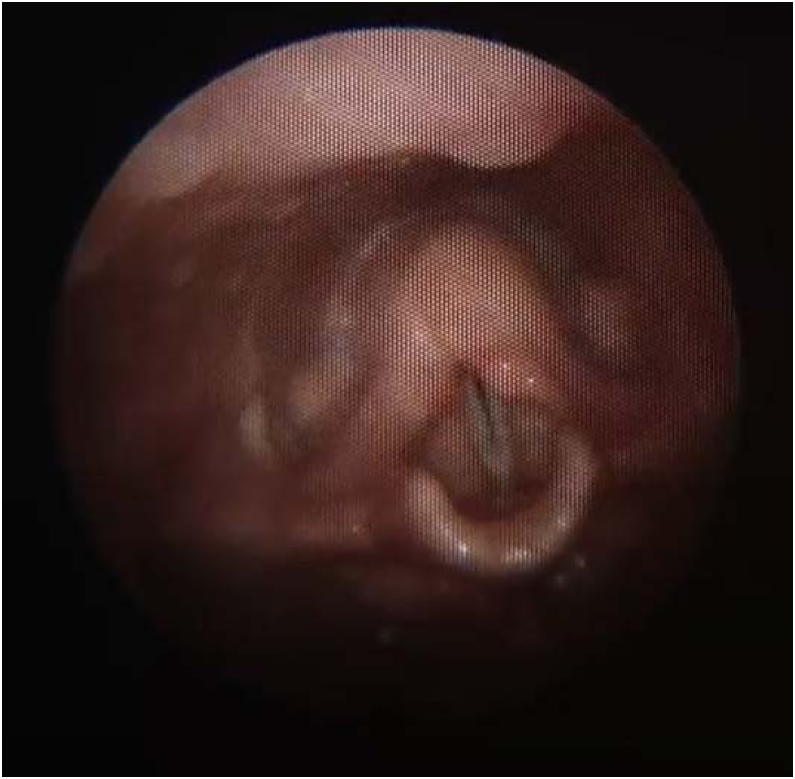
Laryngoscopy after chemo and radiotherapy, showing only mild thickness at the left posterior arytenoid region.

MRI after this treatment showed marked reduction of the lesion in comparison to previous exams (1.3 × 1.0 × 1.0 cm), without metastases. Laryngeal endoscopy showed important reduction of the lesion, restricted to left posterior arytenoid (Fig. 5). PET/CT showed that the reminiscent lesion presented a very low glycolytic metabolism, suggesting absence of neoplasia, and possible a cicatricial tissue following chemo/radiotherapy. After a short-term follow up (two months after completing treatment), the patient remains in clinical remission.

## Discussion

Rhabdomyosarcoma is the most common soft tumor in children, mainly located at the head and neck region, followed by genitourinary tract and extremities.^4^ Our patient presented laryngeal symptoms (progressive hoarseness and dyspnea) due to this well-defined supraglottic nodular lesion. This presentation is extremely unusual, since the sites mainly affected in head and neck region are the orbits, parameningeal sites (nasopharynx, nose, sinuses, temporal bone, pterygopalatine fossa, and infratemporal fossa) and the non-parameningeal sites.^5^ The larynx is affected in less than 3% of head and neck rhabdomyosarcomas^6^ and, according to a systematic review, only 37-cases have been documented, none of them in Brazil.^4^ In the literature, the laryngeal rhabdomyosarcoma is reported to have a better outcome than in other sites at the head and neck, due to early diagnosis and surgical access.^7^

Diagnosis is confirmed by histopathologic evaluation of the tumor specimen. Rhabdomyosarcoma is a solid and dense tumor. Four histological subtypes are described: embryonal, alveolar, pleomorphic and sclerosing, depending on the degree of cell maturation and differentiation.^7^

According to Intergroup Rhabdomyosarcoma Study Group (IRSG) 2011, the preconized treatment is the combination of chemotherapy, surgery (with complete resection while preserving function whenever possible) and local radiotherapy (depending on tumor location, histology and resectability). Therapy should be guided by protocols based on risk-adapted stratification, grounded on clinical, histological, and genetic features.

Follow-up is recommended for at least 5-years after treatment is completed to evaluate recurrence, and until adulthood, to recognize late adverse effects related to therapy, such as thyroid dysfunction when the tumor is located at head or neck.^5^ Patients classified as Low Risk B present 83% of chance of survival without the disease in 5-years and overall survival rate of 95%, according to Intergroup Rhabdomyosarcoma Study Group (IRSG).^8^

## Conclusion

Differential diagnosis of laryngeal lesions is of extreme importance for adequate treatment. It is fundamental to reassure that, despite the fact that benign tumors such as recurrent laryngeal papillomatosis are the commonest lesions observed in this setting, the otorhinolaryngologist should be alert to signs and symptoms that suggest malign tumors. Prompt diagnosis of RMS and other malignant tumors affecting the larynx in children is crucial to rapidly initiate proper oncologic treatment.

## Funding

The authors received no financial support for this article’s research, authorship, or publication.

## Conflicts of interest

The authors declare no conflicts of interest.
